# *Bifidobacterium longum* and *Chlorella sorokiniana* Combination Modulates IFN-γ, IL-10, and SOCS3 in Rotavirus-Infected Cells

**DOI:** 10.3390/ijms25105514

**Published:** 2024-05-18

**Authors:** Felizardo Velderrain-Armenta, Guadalupe González-Ochoa, Patricia Tamez-Guerra, Ricardo Romero-Arguelles, César I. Romo-Sáenz, Ricardo Gomez-Flores, Lilian Flores-Mendoza, Ramona Icedo-García, José G. Soñanez-Organis

**Affiliations:** 1 Department of Chemistry-Biology and Agriculture, Interdisciplinary Faculty of Biology Sciences and Health, University of Sonora, Navojoa C.P. 85880, Mexico; feli_fel@hotmail.com (F.V.-A.); lilian.flores@unison.mx (L.F.-M.); ramona.icedo@unison.mx (R.I.-G.); jose.organis@unison.mx (J.G.S.-O.); 2 Laboratory of Immunology and Virology, Falculty of Biological Sciences, Autonomous University of Nuevo Leon, San Nicolás de los Garza C.P. 66455, Mexico; ricardoromeroarguelles@gmail.com (R.R.-A.); cirscirscirs1989@gmail.com (C.I.R.-S.); ricardo.gomezfl@uanl.edu.com (R.G.-F.)

**Keywords:** rotavirus, *Bifidobacterium*, *Chlorella*, gastroenteritis

## Abstract

Rotavirus is the main cause of acute diarrhea in children up to five years of age. In this regard, probiotics are commonly used to treat or prevent gastroenteritis including viral infections. The anti-rotavirus effect of *Bifidobacterium longum* and *Chlorella sorokiniana*, by reducing viral infectivity and improving IFN-type I response, has been previously reported. The present study aimed to study the effect of *B. longum* and/or *C. sorokiniana* on modulating the antiviral cellular immune response mediated by IFN-γ, IL-10, SOCS3, STAT1, and STAT2 genes in rotavirus-infected cells. To determine the mRNA relative expression of these genes, HT-29 cells were treated with *B. longum* and *C. sorokiniana* alone or in combination, followed by rotavirus infection. In addition, infected cells were treated with *B. longum* and/or *C. sorokiniana*. Cellular RNA was purified, used for cDNA synthesis, and amplified by qPCR. Our results demonstrated that the combination of *B. longum* and *C. sorokiniana* stimulates the antiviral cellular immune response by upregulating IFN-γ and may block pro-inflammatory cytokines by upregulating IL-10 and SOCS3. The results of our study indicated that *B. longum*, *C. sorokiniana*, or their combination improve antiviral cellular immune response and might modulate pro-inflammatory responses.

## 1. Introduction

*Bifidobacterium* species are probiotics commonly used as supplements to improve human health. They are particularly effective in treating or preventing gastrointestinal infections by shortening infectious diarrhea duration and severity [1,2]. The beneficial effect of probiotics is associated with the restoration of intestinal dysbiosis through the production of molecules such as short-chain fatty acids, bacteriocins, and others [1,2,3,4]. Bifidobacteria have also been reported to block infectivity or activate cellular immune responses against viral infections [2,4,5].

The health-beneficial effects of probiotics are limited by microbial load and viability [3]. They are usually combined with prebiotics or microalgae to improve probiotic viability [1,5]. In this regard, the use of the microalga *Chlorella sorokiniana* and *Chlorella vulgaris* has been shown to improve the viability and growth of probiotics, such as *Lactobacillus rhamnosus*, *Lactobacillus acidophilus*, and *Bifidobacterium lactis* [6,7,8]. In addition, *C. sorokiniana* has been shown to improve the viability and shelf life of probiotics, such as *Bifidobacteium longum* and *Lactobacillus plantarum* [5,9]. Furthermore, the microalga *Chlorella* is considered a functional food. It has been associated with antimicrobial activity and is increased in beneficial microorganisms’ population in the intestines, which improves gut health [10].

On the other hand, viruses are a common cause of gastroenteritis worldwide [11]. In virus-infected cells, the first line of defense is the cellular immune response, mediated by interferons (IFNs) [12]. Type I IFNs comprise a large group of molecules within the IFN-α and IFN-β genes that directly induce antiviral response [12,13,14]. In addition, type II interferon gamma (IFN-γ) has antiviral activity and immunomodulatory functions. It has been related to the inhibition of viral infection by transmissible gastroenteritis virus and other viruses [15,16,17,18]. Although type I IFNs and IFN-γ directly activate the expression of interferon-stimulated genes (ISGs) and trigger antiviral activity, IFN-γ induces a different but partially overlapping set of genes [18]. Type I and II IFNs stimulate a significant expression and activation of STAT1, whereas IFN-α and IFN-β stimulate those of STAT2 [19]. STAT proteins mediate the signal transduction of cytokines involved in improving the antiviral response [19]. However, cytokine overstimulation mediated by JAK/STAT’s signaling is associated with pro-inflammatory conditions, which may also cause tissue damage. In this regard, interleukin-10 (IL-10) is a regulatory cytokine that suppresses the immune response by blocking the action of pro-inflammatory cytokines. Suppression of cytokine signaling 3 (SOCS3) is also induced by IL-10 [20,21] and exerts negative feedback regulation on type I IFN and the JAK/STAT signaling pathway [20,22].

Within gastrointestinal pathogens, rotavirus causes acute gastroenteritis in children with symptoms such as watery diarrhea, vomiting, fever, and dehydration [23,24]. Rotavirus belongs to the *Reoviridae* family and possesses an icosahedral shape structure of 75 nm. Rotavirus has three concentric protein layers that surround a segmented genome of 11 fragments of double-stranded RNA (dsRNA). The viral genome encodes the structural proteins VP1-VP4, VP6, and VP7 and the non-structural proteins NSP1 to NSP6 [24]. The viral proteins VP7 and VP4 are part of the rotavirus external layer; VP6, the most abundant protein, is the intermediate layer, and VP2 is the inner layer of the viral particle. In the internal layer, VP1 is also present and functions as a viral polymerase, whereas VP3 is an RNA-capping enzyme [13]. The rotavirus pathogenesis is attributed to NSP1 and NSP4. In infected cells, NSP4 causes Ca^2+^-dependent Cl^−^ secretion across the mammalian small intestinal mucosa, acting as a diarrhea-inducing enterotoxin. Furthermore, NSP1 participates in the evasion of the innate immune response by suppressing the type I IFN response and improving viral replication, hence increasing gastroenteritis severity [24,25,26,27].

Currently available vaccines against rotavirus include the human monovalent G1P [8] vaccine Rotarix^®^ (GlaxoSmithKline Biologicals, Rixensart, Belgium) and the pentavalent human–bovine reassortant vaccine RotaTeq^®^ (Merck & Co., West Point, PA, USA). Vaccines are effective against rotavirus severe diarrhea, but their disadvantages involve their limited distribution and effectiveness in developing countries. Rotavirus is still the main cause of gastroenteritis-associated morbidity and mortality in children worldwide [23]. Although these vaccines offer heterotypic protection against diverse genotypes (not-vaccinal genotypes), the rotavirus strain diversity would have an impact on the efficacy and effectiveness of a vaccine [28]. On the other hand, some reports have shown the effect of anti-rotavirus agents targeting host factors in different stages of viral pathogenesis, but those studies still require further investigation. At present, we lack antiviral agents approved against rotavirus [29].

On the other hand, *Lactobacillus* and *Bifidobacterium* are associated with restored antiviral signaling by up-regulating interferon levels [30,31,32]. Furthermore, microalgae *Chlorella* have demonstrated a beneficial immunomodulatory effect against enteric pathogens [6,7,8]. In previous reports, *Bifidobacterium longum* reduced rotavirus infectivity to 72%, and the combination of *B. longum* and *Chlorella sorokiniana* reduced the viral infectivity to 30%, thus indicating that the antiviral effect of *B. longum* was improved by *C. sorokiniana* [5]. Moreover, *B. longum* induces an in vitro antiviral response mediated by IFN-α in cells infected with rotavirus. The antiviral response induced by *B. longum* and *C. sorokiniana* was shown to be mediated by IFN-α and IFN-β [33]. Although a cellular response by type I IFNs in cells treated with *B. longum* and *C. sorokiniana* in rotavirus-infected cells was observed, the role of type II IFNs, STAT1/STAT2, IL-10, and SOCS3 in the immunomodulatory cellular effect of this probiotic and microalgae is still unknown. This work aimed to study the effect of *B. longum* and *C. sorokiniana* on modulating the antiviral cellular immune response mediated by IFN-γ, IL-10, SOCS3, STAT1, and STAT2 genes in rotavirus-infected cells.

## 2. Results

### 2.1. Rotavirus-Infected Cells

**Figure 1:**
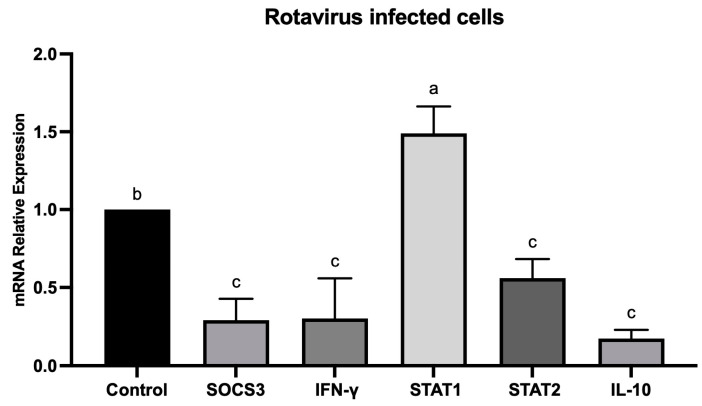
Rotavirus (RV)-infected cell assays. Relative expression level of SOCS3, IFN-γ, STAT1, STAT2, and IL-10 genes in HT-29 cells infected with rotavirus using uninfected HT-29 as the control. Data were analyzed by ANOVA with subsequent Tukey test using GraphPad Prism 10. Different letters indicate statistical significance between treatments. *p* values < 0.05 were considered statistically significant.

The mRNA levels of IFN-γ, IL-10, SOCS3, STAT1, and STAT2 genes were determined in HT-29 cells infected with rotavirus (without probiotic and microalga treatments). The results indicated that the relative expression levels of SOCS3, IFN-γ, STAT2, and IL-10 were lower in rotavirus-infected cells than in non-infected cells, whereas STAT1 was up-regulated compared to the control (non-infected cells) (Figure 1).

### 2.2. B. longum and Rotavirus Assays

**Figure 2:**
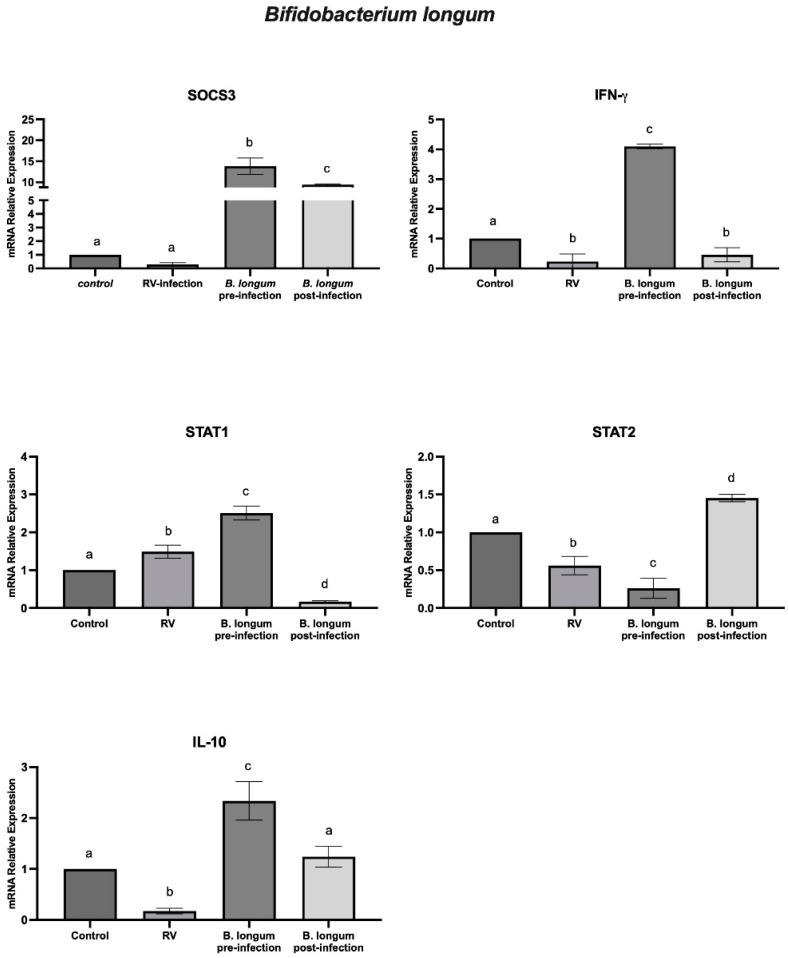
*B. longum* and rotavirus (RV) assays. The relative expression levels of SOCS3, IFN-γ, STAT1, STAT2, and IL-10 genes in HT-29 cells treated with *B. longum* before (pre-infection) and after (post-infection) rotavirus infection were analyzed by ANOVA with subsequent Tukey test using GraphPad Prism 10. Different letters indicate statistical significance between treatments. *p* values < 0.05 were considered statistically significant.

To study the in vitro antiviral effect of *B. longum* in HT-29 cells (their viability was not affected by treatments) in the pre- and post-infection assays with rotavirus, the mRNA levels of IFN-γ, IL-10, SOCS3, STAT1, and STAT2 genes were measured. Our results showed a significant (*p* < 0.05) increase in mRNA of SOCS3, IFN-γ, IL-10, and STAT1 in cells incubated with the probiotic and infected with rotavirus. In addition, in cells infected and treated with the probiotic, we observed a significant (*p* < 0.05) increase in SOCS3, IL-10, and STAT2 relative expression (Figure 2).

### 2.3. C. sorokiniana and Rotavirus Assays

**Figure 3:**
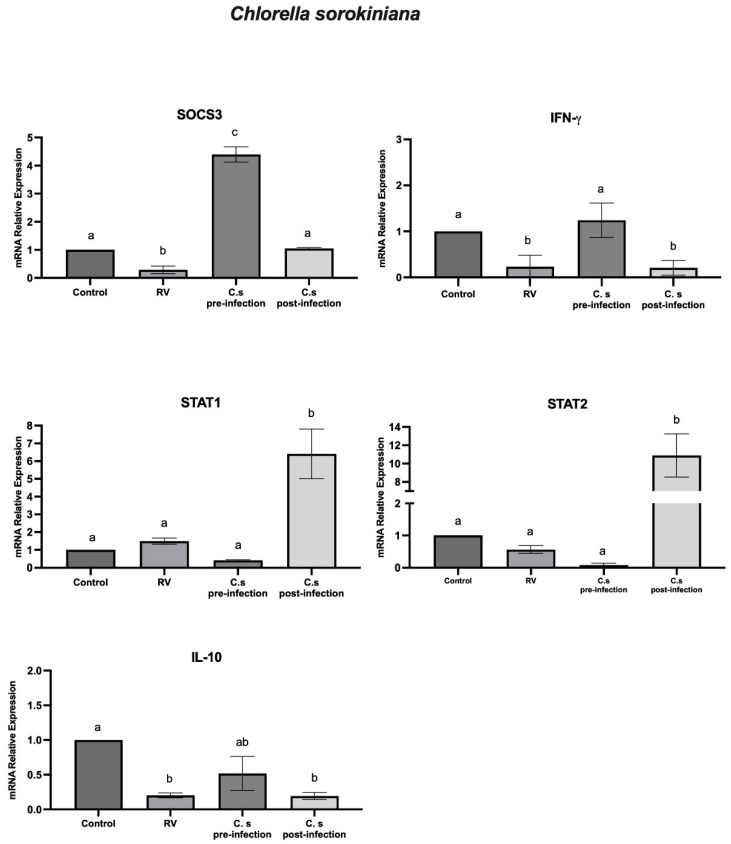
*C. sorokiniana* and rotavirus (RV) assays. The relative expression level of SOCS3, IFN-γ, STAT1, STAT2, and IL-10 genes in HT-29 cells treated with *B. longum* before (pre-infection) and after (post-infection) rotavirus infection. Data were analyzed by ANOVA with subsequent Tukey test using GraphPad Prism 10. Different letters indicate statistical significance between treatments. *p* values < 0.05 were considered statistically significant.

To determine the effect of *C. sorokiniana* in HT-29 cells, we studied the mRNA levels of IFN-γ, IL-10, SOCS3, STAT1, and STAT2 genes in cells incubated with *C. sorokiniana* in the rotavirus pre- and post-infection assays. In cells with *C. sorokiniana* and infected with rotavirus, we observed a significant (*p* < 0.05) increase in mRNA levels of SOCS3 and IFN-γ. Furthermore, in the post-infection assays with rotavirus and *C. sorokiniana,* we observed a significant (*p* < 0.05) relative expression of SOCS3, STAT1, and STAT2. The mRNA level of IL-10 was downregulated in the pre-infection and post-infection assays (Figure 3).

### 2.4. B. longum in Combination with C. sorokiniana and Rotavirus Assays

**Figure 4:**
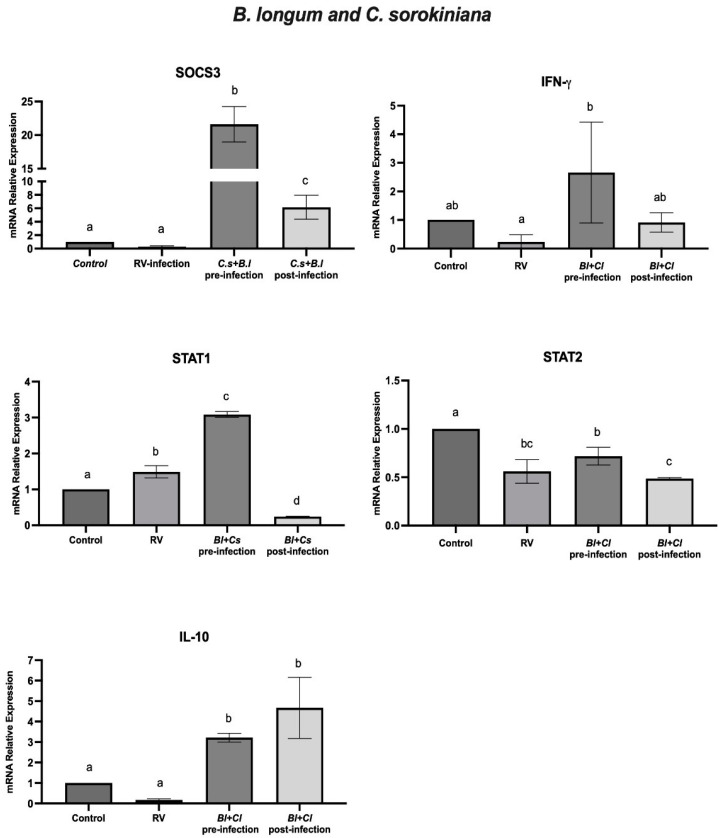
*B. longum* in combination with *C. sorokiniana* and rotavirus (RV) assays. The relative expression level of SOCS3, IFN-γ, STAT1, STAT2, and IL-10 genes in HT-29 cells treated with *B. longum* and *C. sorokiniana* before (pre-infection) and after (post-infection) rotavirus infection. Data were analyzed by ANOVA with subsequent Tukey test using GraphPad Prism 10. Different letters indicate statistical significance between treatments. *p* values < 0.05 were considered statistically significant.

**Figure 5:**
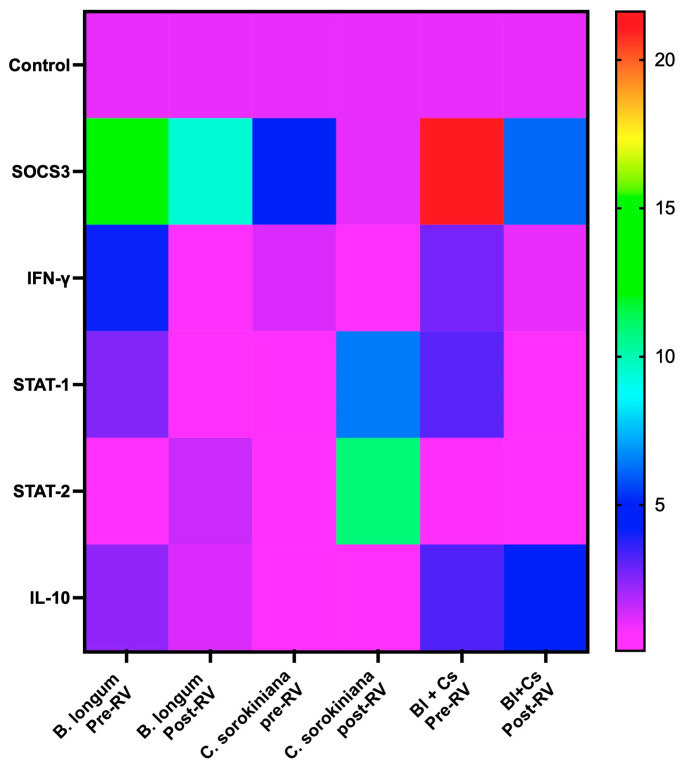
Heat map showing the mRNA expression of SOCS3, IFN-γ, STAT-1, STAT-2, and IL-10 in *B. longum* pre-infected with rotavirus (pre-RV): HT-29 cells infected with RV and treated with *B. longum* (Bl); *B. longum* post-infected with RV (post-RV): HT-29 cells treated with Bl and infected with RV; *C. sorokiniana* pre-RV: HT-29 cells infected with RV and treated with *C. sorokiniana* (Cs); *C. sorokiniana* post-RV: HT-29 cells treated with Cs and infected with RV; Bl + Cs pre-RV: HT-29 cells treated with *B. longum* in combination with *C. sorokiniana* and then infected with RV; Bl + Cs post-RV: HT-29 cells infected with RV and treated with *B. longum* in combination with *C. sorokiniana*. Data were analyzed by three-way ANOVA with subsequent Tukey test using GraphPad Prism 10.

A combination of *B. longum* and *C. sorokiniana* was used to study its effect on the antiviral response in HT-29 by measuring the mRNA levels of IFN-γ, IL-10, SOCS3, STAT1, and STAT2 genes in cells with the combination of *B. longum*/*C. sorokiniana* in the pre- and post-infection assays with rotavirus. In cells incubated with both microorganisms and infected with rotavirus (pre-infection), we observed a significant (*p* < 0.05) upregulation of the mRNA levels of SOCS3, IFN-γ, STAT1, and IL-10. Moreover, in the post-infection assays (cells infected and further treated), the mRNA levels of SOCS3, IL-10, and IFN-γ were significantly (*p* < 0.05) upregulated, whereas STAT1 was downregulated (Figure 4). In Figure 5, a heat map summarizes the mRNA expression of SOCS3, IFN-γ, STAT-1, STAT-2, and IL-10 in cells with *B. longum* and/or *C. sorokiniana* and pre- or post-infected with rotavirus.

## 3. Discussion

Rotavirus is the main cause of acute gastroenteritis in children worldwide [23]. In the present study, we showed that, in HT-29 cells infected with rotavirus, the mRNA relative expression of SOCS3, IFN-γ, and IL-10 was downregulated (*p* < 0.05), but a significant difference with STAT1 and STAT2, as compared with cells without rotavirus infection, was not observed (Figure 1). Previous studies reported that rotavirus block gene expression induced by type I and II IFNs. In addition, rotavirus was associated with STAT1/STAT2 inhibition, which decreased antiviral response in early infection, through the activity of NSP1 [34,35]. Rotavirus protein NSP1 inhibits IFN production by degrading the interferon precursors IRF3, IRF5, and IRF7. It also inhibits NFkB, which decreases IFN-β and cytokine production in infected cells, and IFN signaling by preventing the nuclear accumulation of STAT1/ STAT2 [35,36]. Some other viruses besides rotavirus express proteins associated with cellular immunity evasion [12]. On the other hand, probiotics might modulate antiviral immune responses. In this regard, *Lactobacillus mucosae* and *Bifidobacterium breve* have been associated with restoring antiviral signaling by a positive regulation of interferon levels [33].

In previous reports, we demonstrated that *B. longum* improved monolayer integrity, rotavirus load reduction, and in vitro anti-rotavirus response through IFN-α [33]. In this regard, the probiotics *Lactobacillus* spp. and *Bifidobacterium spp.* have been associated with reduced severity and shorter periods of diarrhea. In addition, in vitro assays with *B. longum* R0175 in porcine intestinal epitheliocyte cells before rotavirus infection showed a preventive effect against rotavirus infection [37]. In this study, we found a significant (*p* < 0.05) increase in the relative expression of SOCS3, IFN-γ, IL-10, and STAT1 in cells with *B. longum* and rotavirus, whereas a higher relative expression of SOCS3 and STAT2 was observed in cells infected with rotavirus and incubated with *B. longum*. In agreement with our analysis of SOCS3, studies in RAW264.7 cells exposed to *Bifidobacterium* species showed increased mRNA levels of SOCS1 or SOCS3. These results indicated that this probiotic may negatively modulate the levels of pro-inflammatory cytokines [20]. Furthermore, other studies have demonstrated the immunomodulatory effect of *Bifidobacterium* species through the up-regulation of IFN-γ and IL-10. Moreover, an anti-inflammatory effect of this probiotic on HT-29 cells has been described via modulation of JAK/STAT [38,39].

On the other hand, *Chlorella* genus is a widely studied microalga for its application in industries such as animal nutrition, pharmaceuticals, and health [6,40,41]. We have previously reported that cells treated with *C. sorokiniana* metabolites caused a significant reduction in rotavirus infectivity [5]. In this regard, *Chlorella* supplementation has shown an effect against hepatitis C by reducing viral load [33,42]. Furthermore, IFN-α relative gene expression was upregulated in cells with *C. sorokiniana* and rotavirus (post-infection assays). Nevertheless, there was a low relative expression of IFN-α in cells infected with rotavirus before the microalgae treatment. Our previous results indicated that *C. sorokiniana* blocked rotavirus infectivity and improved immune cellular response. In order to further study the effect of *C. sorokiniana* in rotavirus-infected cells, we investigated the relative expression of IFN-γ, IL-10, SOCS3, STAT1, and STAT2 genes. Our results indicated that, in HT-29 cells incubated with *C. sorokiniana* followed by rotavirus infection, the relative expression of SOCS3 and IFN-γ significantly increased as compared with that of infected cells without microalgae treatment, whereas in cells infected and treated with *C. sorokiniana*, the relative expression of SOCS3, STAT1, and STAT2 was significantly upregulated. This may indicate that the antiviral response induced by *C. sorokiniana* in rotavirus-infected cells was mediated by IFN-α and IFN-γ in the pre-infection assays and STAT1/STAT2 in the post-infection assays and regulated by SOCS3 in both assays. This result agrees with studies with *C. vulgaris* in mice infected with *Listeria monocytogenes,* which demonstrated an increased production of IFN-γ, thus enhancing intracellular resistance to pathogens [43]. In addition, in a randomized, double-blinded, placebo-controlled trial, a significant increase in IFN-γ was observed after eight weeks of *C. vulgaris* supplement intake (pills) [34].

The microalga *C. sorokiniana* has been related to probiotic viability enhancement and effectiveness against pathogens. *C. sorokiniana* and *C. vulgaris* are also associated with prebiotic activity by stimulating *L. rhamnosus* growth [6], and other species showed potential for improving *L. acidophilus* and *B. lactis* viability in yogurt [7,8]. We previously reported that *C. sorokiniana* improved the viability and anti-rotavirus effect of *B. longum* and *L. plantarum* [5]. In addition, we observed increased IFN-α levels in cells with the probiotic/microalgae after rotavirus infection, whereas IFN-β was elevated in cells infected and treated [33]. In the present study, we also evaluated the effect of *B. longum* and *C. sorokiniana*, including analyses of mRNA relative expression levels of IFN-γ, IL-10, SOCS3, STAT1, and STAT2 genes. Our results indicated that, in HT-29 cells with both microorganisms before rotavirus infection, the relative expression of SOCS3, IFN-γ, STAT1, and IL-10 was significantly (*p* < 0.05) upregulated. Furthermore, in the post-infection assays (cells infected and post-treated), the relative expression of SOCS3, IL-10, and IFN-γ was significantly upregulated. The effect of *B. longum* combined with *C. sorokiniana* against rotavirus in infected cells may be associated with blocking infectivity and inducing an antiviral cellular response mediated by type I and II IFNs.

The effect of the *B. longum*/*C. sorokiniana* combination in cells infected is mediated by the improvement of the cellular immune response. IFN-α, IFN-β, and IFN-γ significantly increase the expression and activation of STAT1, whereas IFN-α and IFN-β increase that of STAT2 [19]. In this regard, we observed an increased level of mRNA expression of IFN-γ and STAT1 in pre-infection with the probiotic alone or with the microalgae, which was associated with an improvement of antiviral cellular response, in comparison with rotavirus-infected cells alone. On the other hand, IL-10 is a regulatory cytokine that suppresses the immune response by blocking the action of pro-inflammatory cytokines. In addition, SOCS3 is induced by IL-10 [20,21]. SOCS3 exerts negative feedback regulation on IFN type I and the JAK/STAT signaling pathway [20,30]. In the present study, we observed an increase in the relative expression of IL-10 and SOCS3 in the pre- and post-infection assays with *B. longum* and *C. sorokiniana* as compared with rotavirus-infected cells without treatments, which may indicate that the protective effect of this probiotic and the microalga was also induced by IL-10 and SOCS3 by downregulating a pro-inflammatory response in rotavirus-infected cells. In this regard, the protective effect of *B. longum* in rotavirus-infected cells was improved with the combination of *C. sorokiniana*. Although some limitations of this study are that we only studied the in vitro assays and mRNA relative expression of SOCS3, IFN-γ, STAT-1, STAT-2, and IL-10, our results are consistent with the protective effect of *B. longum* and *C. sorokiniana* in rotavirus-infected cells and with previous reports of beneficial effects of probiotic and microalga against gastrointestinal pathogens [33,37,38,39,42].

## 4. Materials and Methods

### 4.1. Cells

HT-29 human colon tumor cells (ATCC HTB-38) were used in the assays with *B. longum* and *C. sorokiniana* in rotavirus-infected cells. MA104 rhesus monkey epithelial cells (ATCC CRL-2378) were used for rotavirus propagation and microtitration. HT-29 and MA104 cells were grown in RPMI-1640 and DMEM culture media (Gibco, Grand Island, NY, USA), respectively, and were supplemented with 5% fetal bovine serum (FBS; Mediatech Inc., Corning, NY, USA), 1% antibiotic and antimycotic solution (Caisson Laboratories, Smithfield, UT, USA), and 2 mM L-glutamine, and incubated at 37 °C and 5% CO_2_.

### 4.2. Rotavirus Strain and Viral Titration

MA104 cells were infected with the human Rotavirus strain Wa for propagation and viral titration as follows. Rotavirus was activated with 10 µg/mL trypsin-EDTA solution 10X (Sigma-Aldrich, St. Louis, MO, USA) and incubated at 37 °C for 30 min, after which activated viruses were incubated with MA104 cells at 37 °C in 5% CO_2_ for one hour. Next, the inoculum was replaced with DMEM, and the cells were incubated at 37 °C in 5% CO_2_ for 24 h or until the monolayer lysis was observed. The viral lysates were stored at −20 °C. Rotavirus focus-forming units per mL (FFU/mL) were determined by an immunoperoxidase assay, as previously reported [44]. In brief, rotavirus lysates were used to infect MA104 cells and incubated at 37 °C in 5% CO_2_ for 14 h. After incubation, the cellular monolayer was washed twice with PBS-Ca^2+^, fixed with cold acetone-PBS (80% to 20%), and incubated for 45 min at room temperature. Next, the monolayer was washed twice with PBS-Ca^2+^ and incubated for one hour at 37 °C with the primary anti-rotavirus antibodies (Invitrogen, Carlsbad, CA, USA). After incubation, the cell monolayers were washed twice with PBS-Ca^2+^ and incubated for one hour at 37 °C with horseradish peroxidase (HRP)-anti-sheep IgG conjugate (Invitrogen), followed by incubation with the substrate (0.1 M sodium acetate buffer pH 5.0, 0.64 mg/mL aminoethyl carbazole (Sigma-Aldrich), and 0.36% hydrogen peroxide) for 15 min at 37 °C. After incubation, the reaction was stopped with three washes of water. FFU/mL was calculated as follows: FFU/mL = 20 (microscope objective) × 5.5 (well diameter) × average number of foci (duplicate determinations; 100 to 200 foci/well) × dilution (foci count). The number of viral particles per cell (MOI) was calculated with the number of viral particles used (FFU/mL) per well divided by the number of cells originally seeded in the well. Rotavirus MOI was 0.1 in each assay.

### 4.3. Probiotic

*Bifidobacterium longum* strain (ATCC^®^ 15707) was grown on MPT-broth medium (5.0 g glucose, 10 g casein digest peptone, 0.50 g sodium chloride, 2.5 g yeast extract, 0.05 g ascorbic acid, 0.15 g potassium phosphate monobasic, 0.25 g potassium phosphate dibasic, 0.5 g L-cysteine hydrochloride, and 0.124 mg ferric ammonium citrate) [45]. Five milliliters was aliquoted in 13 × 100 mm borosilicate glass tubes and autoclaved for 15 min at 121 °C and 15 lbs pressure. The sterile MPT-broth was stored at −20 °C until use. *Bifidobacterium longum* was inoculated on MTP-broth medium and incubated at 37 °C. Colony-forming units (CFU) per milliliter were calculated by serial dilutions, and the bacterial inoculum for each assay was adjusted to 1 × 10^6^ CFU/mL.

### 4.4. C. sorokiniana

The microalga *C. sorokiniana* was collected in the San Juan River in Cadereyta, Nuevo León, México [5,46]. It was grown in L-carnitine (LC) nutrient solution (5 mM KNO_3_, 1 mM KH_2_ PO_4_, 2 mM MgSO_4_·7H_2_O, 6.25 mM Ca(NO_3_)_2_·4H_2_ O, 46μM H_3_ BO_3_, 9.15 μM MnCl_2_·4H_2_O, 765 nM ZnSO_4_·7H_2_ O, 320 nM CuSO_4_·5H_2_O, 15 nM (NH_4_)6Mo_7_O_24_·4H_2_O, 20 μM FeSO_4_·7H_2_O, and 20 μM Na_2_ EDTA) at 25 °C and 120 rpm under continuous light at 1400 lumens for 12 d [47].

### 4.5. Cellular Viability Assay

To demonstrate that treatments without rotavirus do not alter cellular viability, we determined that of HT-29 cells incubated with *B. longum* and/or *C. sorokiniana* by the colorimetric 3-(4, 5-dimethylthiazol-2-yl)-2, 5-diphenyltetrazolium bromide (MTT) reduction assay. For this, HT-29 cells were incubated with *C. sorokiniana* and/or *B. longum* in RPMI-1640 medium without FBS for 24 h at 37 °C and 5% CO_2_ in 95% air, after which they were washed twice with PBS, and 20 µL of MTT (Sigma-Aldrich; 5 mg/mL final concentration) was added to the cells and incubated for three additional hours. MTT was then replaced by 10 µL of dimethyl sulfoxide (DMSO; Sigma-Aldrich) and incubated for three minutes under continuous shaking. Optical densities were then determined in a microplate reader (Multiskan GO, Thermo Fisher Scientific Inc., San Jose, CA, USA) at 570 nm [48].

### 4.6. B. longum, C. sorokiniana, and Rotavirus Assays

*B. longum*, *C. sorokiniana,* and their combination were used to treat rotavirus-infected cells. In the pre-infection assays, the cells were treated and post-infected with rotavirus as follows: HT-29 cells were treated with probiotics (1 × 10^6^ CFU/mL), the microalgae biomass (1 × 10^6^ cells/mL), or their combination for 24 h, after which they were infected with rotavirus (MOI 0.1) for one hour at 37 °C and 5% CO_2_ in DMEM without FBS. Next, the inoculum was replaced with DMEM without FBS and incubated at 37 °C in 5% CO_2_ for 24 h. Lysates were then stored at −20 °C until use. In the post-infection assays, the cells were first infected with rotavirus and post-treated with the probiotic and/or microalga. For this, HT-29 cells were infected with rotavirus (MOI 0.1) for one hour at 37 °C and 5% CO_2_ in DMEM without FBS, followed by treatment with *B. longum* (1 × 10^6^ CFU/mL), *Chlorella sorokiniana* (1 × 10^6^ cells/mL), or their combination for 24 h. After this, the cells were stored at 20 °C until viral RNA purification and qPCR assays.

### 4.7. qPCR Assay

Total RNA extraction from rotavirus-infected cell lysates and/or treated with *C. sorokiniana* and/or *B. longum* was performed by the Trizol method (Life Technologies, Rockville, MD, USA). Total RNA was used as a template for cDNA synthesis (High-Capacity cDNA Reverse Transcription; Applied Biosystems, Foster City, CA, USA). Relative expression of IFN-γ, IL-10, SOCS3, STAT1, and STAT2 genes was determined by qPCR using PGK-1 as an endogenous gene (Table 1). Reactions were developed with the Sensi FAST SYBER Lo-ROX Kit (Bioline, London, UK), following the manufacturer’s instructions. qPCR conditions were 95 °C for 5 min, 45 cycles at 58 °C for 5 s, and 60 °C for 10 s. Gene relative expression was calculated using the 2^(−ΔΔCt)^ method (Applied Biosystems).

### 4.8. Statistical Analysis

In each assay, three independent experiments were performed, and the results were reported as mean ± SD. Statistical analysis was calculated by the one-way ANOVA or three-way ANOVA and Tukey’s multiple comparisons or Kruskal–Wallis and Dunn’s multiple comparison tests using GraphPad Prism 10 (GraphPad Software Inc., San Diego, CA, USA). *p* values < 0.05 were considered statistically significant.

## 5. Conclusions

In this study, we observed that the probiotic *B. longum* increased the relative expression of SOCS3, IFN-γ, IL-10, and STAT1 in cells with *B. longum* and infected with rotavirus and SOCS3 and STAT2 in cells infected with rotavirus and incubated with *B. longum*. On the other hand, in the pre-infection assays with *C. sorokiniana*, the mRNA levels of SOCS3 and IFN-γ were increased, whereas in the post-infection assays, SOCS3, STAT1, and STAT2 were upregulated. Additionally, *B. longum* and *C. sorokiniana* improved the antiviral cellular immune response by upregulating SOCS3 and IFN-γ in the pre- and post-infection assays. This combination may block pro-inflammatory cytokines by upregulating IL-10 and SOCS3. Our results indicated that *B. longum*, *C. sorokiniana*, or their combination improve antiviral cellular immune response and might modulate pro-inflammatory responses. Although further research is needed, supplementation with probiotics such as *Bifidobacterium* and the microalgae *Chlorella* might be beneficial and become an alternative in the prevention or treatment of rotavirus gastroenteritis.

## Figures and Tables

**Table 1:** qPCR primer sequences.

Primer Name	Primer Sequences (5′ to 3′)		Product Length	Reference
	Fwd	Rev		
SOCS3	5′-ACA ATC TGC CTC AAT CAC TCT G-3′	5′-TTG ACT TGG ATT GGG ATT TTG-3′	129	[49]
IFN-γ	5′-GGC ATT TTG AAG AAT TGG AAA G-3′	5′-TTT GGA TGC TCT GGT CAT CTT-3′	112	[49]
STAT1	5′-GAT CGC TTG CCC AAC TCT TG-3′	5′- ACT GTG ACA TCC TTG GGC TG-3′	198	[50]
STAT2	5′-GGC AGC GAA TCA CTC AAA GC-3′	5′-CACCAGAGTCAAGAAGCCGA-3′	159	[50]
IL-10	5′-TGG AGC AGG TGA AGA ATG-3′	5′-ATA GAA GCC TAC ATG ACA-3′	105	[49]
PGK1	5′-GAG ATG ATT ATT GGT GGT GGA A-3′	5′-AGT CAA CAG GCA AGG TAA TC-3′	160	[51]

## Data Availability

The data presented in this study are available on request from the corresponding author.
